# Delamination Behaviour of Embedded Polymeric Sensor and Actuator Carrier Layers in Epoxy Based CFRP Laminates—A Study of Energy Release Rates

**DOI:** 10.3390/polym13223926

**Published:** 2021-11-13

**Authors:** Andreas Hornig, Anja Winkler, Eric Bauerfeind, Maik Gude, Niels Modler

**Affiliations:** 1 Institute of Lightweight Engineering and Polymer Technology (ILK), Technische Universität Dresden, Holbeinstraße 3, 01307 Dresden, Germany; anja.winkler@tu-dresden.de (A.W.); maik.gude@tu-dresden.de (M.G.); niels.modler@tu-dresden.de (N.M.); 2 AB Elektronik Sachsen GmbH, Salzstraße 3, 01774 Klingenberg, Germany; eric.bauerfeind@ab-sachsen.de

**Keywords:** sensor embedding, carrier foil, function-integrative composites, integrated sensor systems, intelligent composites, delamination behaviour

## Abstract

Fiber reinforced composites combine low density with high specific mechanical properties and thus became indispensable for today’s lightweight applications. In particular, carbon fibre reinforced plastic (CFRP) is broadly used for aerospace components. However, damage and failure behaviour, especially for complex fibre reinforcement set-ups and under impact loading conditions, are still not fully understood yet. Therefore, relatively large margins of safety are currently used for designing high-performance materials and structures. Technologies to functionalise the materials enabling the monitoring of the structures and thus avoiding critical conditions are considered to be key to overcoming these drawbacks. For this, sensors and actuators are bonded to the surface of the composite structures or are integrated into the composite lay-up. In case of integration, the impact on the mechanical properties of the composite materials needs to be understood in detail. Additional elements may disturb the composite structure, impeding the direct connection of the composite layers and implying the risk of reducing the interlaminar integrity by means of a lower delamination resistance. In the presented study, the possibility of adjusting the interface between the integrated actuator and sensor layers to the composite layers is investigated. Different polymer layer combinations integrated into carbon fibre reinforced composite layups are compared with respect to their interlaminar critical energy release rates $G_{Ic}$ and $G_{IIc}$. A standard aerospace unidirectionally reinforced (UD) CFRP prepreg material was used as reference material configuration. The investigations show that it is possible to enhance the mechanical properties, especially the interlaminar energy release rate by using multilayered sensor–actuator layers with Polyimide (PI) outer layers and layers with low shear stiffness in between.

## 1. Introduction

Against the backdrop of the scarcity and rising cost of natural resources, the optimization of material and energy efficiency is increasingly coming to the fore of science and industry in technology, more than ever before. Advanced lightweight design concepts are developed for production systems and manufacturing technologies as well as for the use and operation of structures and components. High energy saving potentials with simultaneous pollutant reduction promise material-efficient lightweight solutions with a considerable weight reduction of moving components and systems. In this context, fibre-reinforced composites offer the possibility of adapting the material properties to the specific requirements regarding mechanical [1] and other functionalities, such as appropriate tribological [2] or even ecological compatibility [3]. The relatively new group of textile-reinforced plastics offers particular advantages for complex applications, especially in the fields of aerospace, automotive, marine and mechanical engineering, medical technology and sports equipment [4,5,6]. In this context, such parts were further modified by adhesively bonding of additional functional elements to the composite structure or integrating them into the composite lay-up [7,8,9,10,11,12,13,14]. In most cases, these elements represent sensors, actuators, conductive paths, and small electronic elements. They can be used for different purposes, once they enable the monitoring of the composite structures and allow a forecast of damage propagation or failure [10,15,16].

The second purpose is to use these elements as an internal measurement system for several tasks, e.g., temperature, strain or impact detection [11,12,13,14]. In most cases, the functional elements are not directly embeddable in the lay-up. With regard to electrical insulation and high positional accuracy, these elements can be pre-assembled to functional layers, consisting of electrical insulating cover layers and the functional elements [17,18,19]. In this context, textile-reinforced plastics show decisive advantages compared to conventional construction materials. Primarily, they consist of a layered lay-up, enabling a relatively easy insertion of additional layers. The second main advantage is that they only need very low manufacturing temperatures compared to metals. Thus, a wide variety of modern flat functional elements, such as sensors, actuators, antennas and generators can be integrated directly into the manufacturing process of the textile-reinforced components, which enables further weight and performance advantages of the resulting light-weight system [10].

However, the opportunities offered by this modern combination of materials also present new challenges that the fibre composite engineer has to face. This also includes manufacturing-specific aspects such as the integration of sensor and actuator elements into the structure of fibre composite components and its influence on the manufacturing or design process as well as on the mechanical properties [9,20,21]. In this context, several studies were made with regard to designing suitable smart components [9,22,23], adapted manufacturing technologies for function-integrative structures [17,24] and their experimental characterization [15,25,26]. Common materials are composites of glass fiber reinforced thermosets (mainly epoxy resins) or thermoplastics (e.g., polyamides, polypropylene). In many works, the elements are integrated directly into the composite or adhesively bonded to it. Here, the matrix systems used and the cladding layers of the functional elements themselves act as insulation material. If additional carrier or insulating films are used, these are usually thin films of polyimide e.g., in the case of the SMART Layer™ technology [18,19] or embeddable sensor layers [23].

In particular, the use of CFRPs yields further requirements. The reason for this is in particular the electrical conductivity of the carbon fibers used. Therefore, suitable concepts for the electrical insulation of the integrated elements (e.g., sensors, actuators, conductive paths) are required. Electrical and electronic components and especially the electrical contacting areas, e.g., for connecting the power supply or external measuring devices, have to be electrically insulated appropriately. Studies on the integration of large insulation layers and their impact on the composite properties of CFRPs are only marginally described in literature [18,19,21,27,28].

This paper deals with the use of large and composite adapted active layers enabling proper electrical insulation of all elements to the CFRPs as well as high mechanical properties of such function-integrative composites. It presents investigations for the integration of different plastic films, in the function of an electrical insulation layer, and the characterization of their influence on the mechanical properties of the composite material. The analysis of the modified composites is based on suitable methods for evaluation and validation of mechanical properties and aspires to point out the limits of the corresponding material configurations. Particular attention is paid to minimizing the influence on the mechanical and interlaminar properties of the modified CFRP composites.

## 2. Embedded Polymer Carrier Layers

### 2.1. Investigated Material and Embedding Configurations

To investigate the influence of different polymeric layers integrated in CFRP composite panels, different setups are conceptualized, manufactured and tested. The experimental investigations aim towards a characterization of the bonding behaviour and are based on fracture mechanical experiments (see Section 3). This requires a crack initiation which is realised in all investigated configurations by the use of Polytetraflourethylene (PTFE) foil insertion, which acts as the crack initiation layer. Different polymeric materials are chosen to investigate their embedding behaviour in varying lay-up configurations. The following engineering polymeric foils are considered:

- Polyimide (PI),
- Polyether-Ether-Ketone (PEEK),
- Polyamide (PA),
- Polyetherimide (PEI),
- additional Polyolefine (PO) layer.

These potential materials feature a sufficiently high heat resistance and can therefore resist the CFRP manufacturing process of the composite material undamaged. Additionally, they fulfill the requirements of electrical insulation for sensors, actuators and conductive path integration.

**Figure 1:**
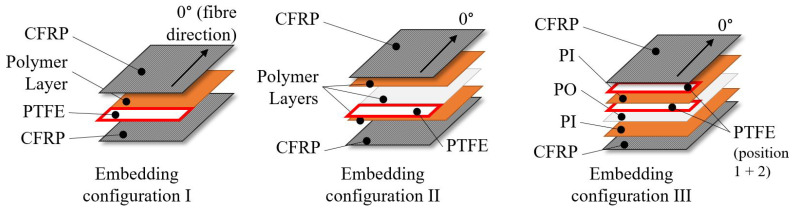
Illustration of investigated embedding configurations.

For the investigations, a three-phased approach with corresponding embedding configurations I–III and systematically varying composite lay-up configurations (Figure 1) is chosen:

- Embedding configuration I: The polymer layers are positioned in the mid plane of the composite lay-up to down select appropriate polymer layer materials.
- Embedding configuration II: Additional Polyolefine (PO) adhesive agent layers in combination with the PI layers are investigated to further enhance delamination resistance.
- Embedding configuration III: A combination of PI and PO layers are embedded in the mid plane of the CFRP lay up. PTFE crack initiation is positioned either between the PO and PI layer or the PI and CFRP layer to investigate the bonding behaviour of PI and PO in detail.

The investigated material and embedding configurations are summarised in Table 1 and illustrated in Figure 1.

### 2.2. Manufacturing of CFRP Panels with Embedded Layers and Specimen Preparation

The CFRP panels are manufactured by hot pressing technology. Standard aerospace UD prepreg HexPly^®^ 8552 is used for configuration I and II (single layer thickness 130 μm, 30 layers). CYCOM^®^ 977-2 UD CFRP-prepreg is used for configuration III and a reference configuration (layer thickness 184 μm, 20 layers). The fibre direction is chosen for the crack propagation direction Figure 1. In total, 10 panels with dimensions of 295 mm × 295 mm × 3.8 mm for each the 10 investigated material configurations (Table 1) are manufactured.

A CNC cutter is used to cut the layers to size in an automated manner. Afterwards, the prepreg and polymer layers are stacked and pre-compacted manually. The consolidation is performed by a heated compressing and a hot pressing mould under defined pressure and temperature conditions according to the manufacturing specifications of the prepreg materials: compacting under vacuum and 7 bar pressure, curing at 110 ${}^{\circ }$C for 60 min and postcuring at 180 ${}^{\circ }$C for 120 min, both under vacuum and pressure. After consolidation and demoulding, the panels are water jet cut into the final specimen configuration (see Section 3).

**Figure 2:**
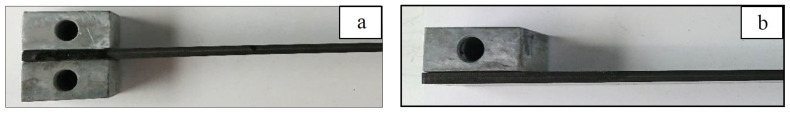
Loading block configuration for (**a**) DCB and (**b**) ELS specimens.

To examine the delamination behaviour, Double-Cantilever Beam (DCB) and End-Loaded Split (ELS) specimens are prepared. The load application to these specimens is realised by adhesively bonded aluminium loading blocks (Figure 2). To ensure an optimal bonding between CFRP and aluminium, the surfaces of both joining partners were pre-treated before the adhesive was applied. The joining surfaces of the CFRP test specimens were first ground using sandpaper with a grain size of 320P. After cleaning with acetone, both the aluminium blocks and the CFRP joining surfaces were blasted with a glass bead size of 40–70 μm. Subsequently, the joining partners are manually joined using a Cyanoacrylate based adhesive that cures at ambient temperature.

## 3. Determination of Delamination Characteristics

### 3.1. Experimental Programme and Setup

Two different experimental procedures are employed to characterize the impact of the embedded polymer layers on the interlaminar fracture characteristics. Mode I critical energy release rates $G_{IC}$ are determined using the DCB test based on [29], whereas the ELS test is used for mode II critical energy release rates $G_{IIc}$ based on [30]. Pre-cracks of 70 mm are realised by embedded PTFE foils in the midplane (see Figure 1). In total, 80 tests are performed (8 per material configuration 1–10, Table 1). An overview is given in Table 2. All experiments are performed under displacement control at a constant rate of 10 mm/min and standard climate conditions (23 ${}^{\circ }$C ± 2 ${}^{\circ }$C, 50 ± 5% relative humidity).

**Figure 3:**
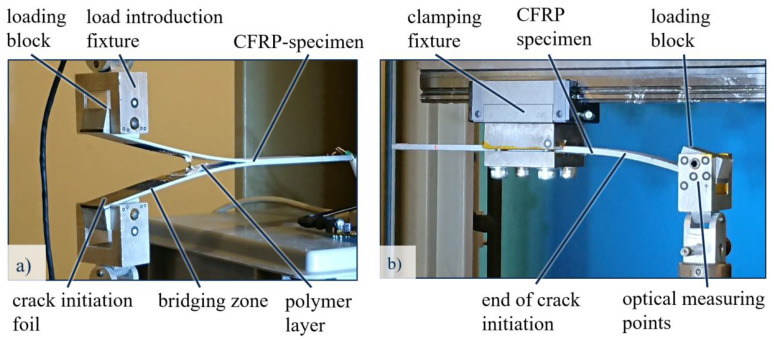
Experimental setup for (**a**) Mode I DCB and (**b**) Mode II ELS Testing.

The crack-opening load is applied to the Mode I DCB specimen perpendicular to the plane of delamination propagation, through loading blocks (Figure 3a). The onset of stable delamination growth is monitored and the delamination initiation and propagation readings are recorded.

For Mode II ELS tests, specimens are clamped in a way that the crack initiation side is connected to the tensile testing machine and the opposite side is fixed in a clamping with a lateral degree of freedom and a free length of 110 mm. Load is applied in thickness direction of the specimen, perpendicular to the plane of delamination (Figure 3b). The upper and lower halves of the specimen are displaced from each other during loading, which drives the crack forward.

Besides the analysis of the force–displacement readings from the universal testing machine ZWICK Z250 (10 kN load cell with a measuring accuracy of ±0.2%), a visual detection of crack propagation has been realised using the digital image correlation (DIC) system ARAMIS 5M (GOM mbH) for both modes I and II. This procedure is explained in detail in [31].

### 3.2. Data Analysis

**Figure 4:**
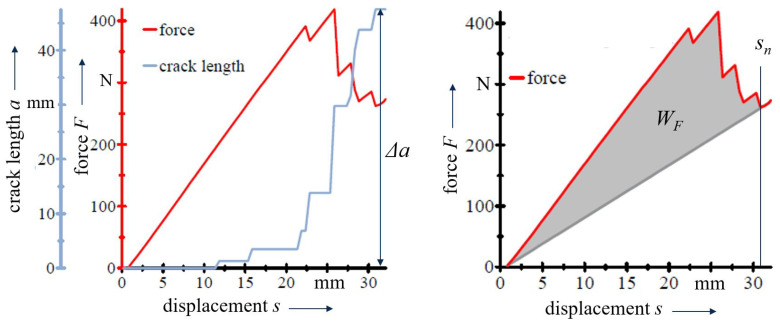
Analysis of energy release rate values based on force–displacement results and synchronous crack length measurements.

The critical energy release rates $G_{Ic}$ and $G_{IIc}$ for the respective modes I and II characterise the delamination resistance of the CFRP. For Mode I and Mode II crack opening, the according $G_{Ic}$ and $G_{IIc}$ values are determined in the DCB and ELS experiments, where the applied load forces the crack to propagate. In both setups, an energy based approach is used in combination with the visual crack length detection based on DIC. The energy release rates are calculated by relating the fracture work $W_{F}$ to the generated fracture surface A=B·Δa:

(1) $$G_{c}=\frac{W_{F}}{B\cdot \Delta a}\;,$$

with *B* denoting the constant specimen width and Δa the propagated crack length [32,33]. Based on the force–displacement (*F*-*s*) results, $W_{F}$ is assumed to be the result of the system’s total energy less the elastic deformation energy (Figure 4):

(2) $$W_{F}=\int_{0}^{s_{n}}\;F\;ds-\frac{1}{2}F\left(s_{n}\right)\cdot s_{n}\;,$$

with $s_{n}$ denoting the evaluation displacement.

**Figure 5:**
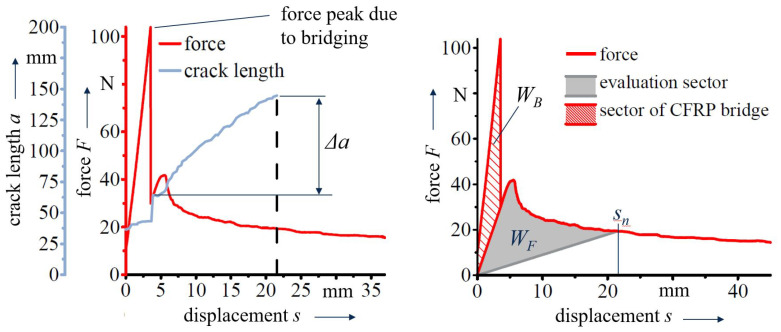
Analysis of energy release rate values accounting for bridging zones.

In some specimens, a bridging zone is observed, caused by a misalignment of the polymer layers during manufacturing. This zone is characterised by a gap between crack initiation foil and the polymer layer, subsequently resulting in a direct contact of the CFRP-layers. In the *F*-*s*-curves, it is indicated by a distinct force peak before crack propagation is observable. The peak is similar over different tested material configurations (max. deviation of ±2.5%). In these cases, the fracture work forcing the crack through the bridging zone $W_{B}$ is excluded from energy release rate calculation (Figure 5):

(3) $$W_{F}=W_{total}-W_{B}.$$

## 4. Results and Discussion

The results of the experiments for material configuration 1–10 (Table 1) are summarized in Table 3, where the mean values for $G_{Ic}$ and $G_{IIc}$ are listed with the respective standard deviations *S*. In addition, the deviations from the reference configuration 10 (crack initiation between CFRP layers without an additional embedded polymer layer) $\Delta_{ref}$ are given by

(4) $$\Delta_{ref}=\frac{G_{I/IIc}-G_{I/IIc}^{ref}}{G_{I/IIc}^{ref}}\cdot 100\%\;,$$

with $G_{I/IIc}^{ref}$ denoting the energy release rate of the reference configuration.

### 4.1. Reference Configuration

The determined $G_{Ic}$ value of 0.28 kJ/m${}^{2}$ for the reference configuration (material configuration 10, Table 1) without any polymer layer embedding based on the CYCOM^®^ 977-2 material (used for embedding configuration III) are in good correlation to the findings in [34], where 0.25 kJ/m${}^{2}$ are reported for the HexPly^®^ 8552 material (used for embedding configurations I and II) and emphasises a qualitative comparability of the results for the three investigated embedding configurations. A range from 0.31 kJ/m${}^{2}$ to 0.59 kJ/m${}^{2}$ was identified in [35] for delamination propagation between layers of different fibre orientations. In [36] 0.24 kJ/m${}^{2}$ for a HexPly^®^, 6376-NCHR was reported. The strong impact of fibre orientations and subsequent fracture surface waviness are also reported in [37], where values of 0.86 kJ/m${}^{2}$ for 0° and 0.51 kJ/m${}^{2}$ for 90° crack propagation direction were determined for a multi-layered knitted fabric epoxy CFRP material. [38] provides a value of 0.35 kJ/m${}^{2}$ for the 977-2 material.

The $G_{IIc}$ value amounts to 0.73 kJ/m${}^{2}$, which is in correlation to a reported value of 0.59 kJ/m${}^{2}$ for the CYCOM^®^ 977-2 in [38]. In contrast to Mode I, where the DCB experimental procedure is consistently used, different methods besides the ELS setup in this study such as End Notched Flexure (ENF), Stabilized End Notched Flexure (SENF), Centre Notched Flexure (CNS), and Four point End Notched Flexure (4ENF) can be used to characterise Mode II delamination behaviour, and comparable values for the Mode II energy release rates are rarely published. As reported in [31], crack tip position measurement and analysis are particularly challenging. In addition, a pronounced ductile-like fracture behaviour was observed leading to a very limited number of specimens where crack propagation could be observed. This is reflected by the rather poor statistical representation of the results. In [34], a value of 1.759 kJ/m${}^{2}$ was determined. The very large deviation to the presented values can be attributed to a different experimental setup, where crack tip measurement could not be supported by DIC.

It should be emphasized that published results on Mode I and especially Mode II crack propagation properties often differ significantly and exhibit considerable standard deviations. This is attributed to the used testing and evaluation methods as well as crack propagation directions and investigated material configurations, but also on testing laboratories and research groups. This aspect has been already identified for both Modes in [32,33], where the results of different testing laboratories and analysis methods are compared. This is also supported by the findings in [37], where a rather broad range of 2.60 to 9.09 kJ/m${}^{2}$ for 0° and 1.93 to 7.33 kJ/m${}^{2}$ for 90° crack propagation direction was identified. The presented findings of this paper should therefore serve primarily in a qualitative manner to compare the investigated configurations among each other. In this respect, embedding configurations I and II (Figure 1) has been performed as a comparative study to identify the most promising foil material and stacking set-up, whereas embedding configuration III serves as a more detailed study and set-up optimization.

### 4.2. Embedding Configuration I

**Figure 6:**
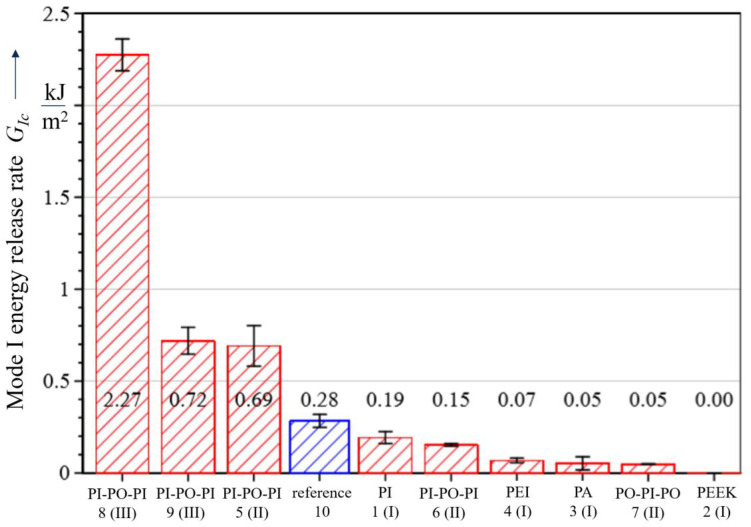
Mode I energy release rates.

**Figure 7:**
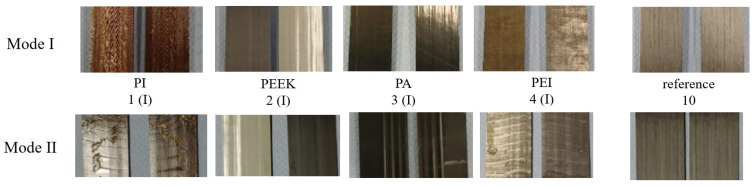
Representative fracture surfaces resulting from Mode I (DCB) and Mode II (ELS) testing for embedding configuration I and reference configuration.

The considered material configurations 1–4 (Table 1) with single embedded polymer layers lead to reduced $G_{I}c$ values in comparison with the reference material configuration 10 and hence to reduced interlaminar properties as displayed in Figure 6. Best results are achieved by the PI polymer layer with a value of 0.19 kJ/m${}^{2}$ and a 32% decrease compared to the reference configuration (Table 3). In addition, a cohesive type of fracture can be observed in configuration 1, where the crack is propagating through the PI layer and the remaining parts of the polymer are still attached to the CFRP on both sides of the separated specimen (Figure 7). The results for PEI with −76% and PA with −82% reduction indicate a more disadvantageous behaviour with an adhesive fracture pattern between polymer foil and CFRP.

**Figure 8:**
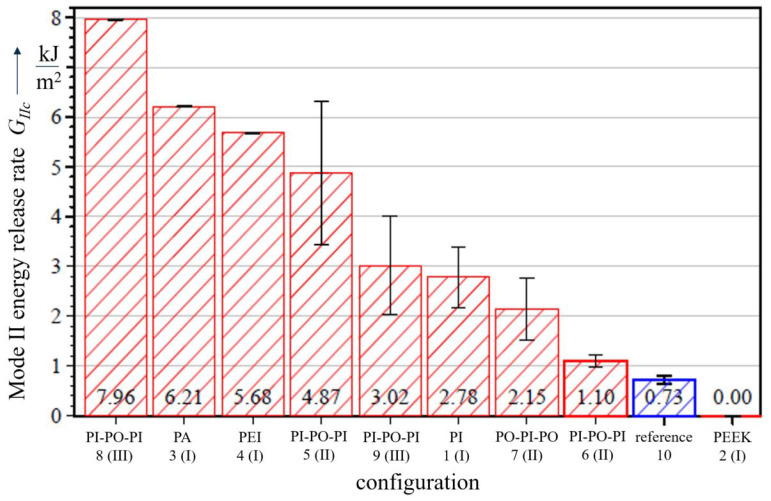
Mode II energy release rates.

In contrast to the Mode I results, none of the investigated configurations (except the PEEK configuration) revealed interface weakening effects under Mode II loading conditions (Figure 8) and a trend of an improved delamination resistance can be clearly observed. This is also backed up by the fracture behaviour, where, for the PI and PEI configurations, a cohesive type of fracture is identified with polymer foil remnants on both fracture sides. However, in case of the PA configuration, cohesive failure is observed. A special case for both Mode I and II loading is the PEEK configuration, which does not show any bonding capabilities and is therefore not considered in further discussions.

The results imply that a structurally weakening effect can be expected under Mode I loading when the considered polymers are embedded in this ’plain’ manner, where these configurations would promote delamination failure. In addition, Mode II appears to be of secondary significance, since no negative impact on the delamination resistance is observed.

### 4.3. Embedding Configuration II

**Figure 9:**
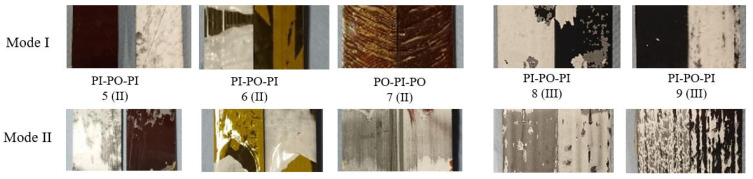
Representative fracture surfaces resulting from Mode I (DCB) and Mode II (ELS) testing for embedding configurations II and III.

Based on the findings in the first testing phase with configuration I, PI-polymer layers are chosen for subsequent embedding investigations, whereas PEI and PA did not qualify for further considerations. Aiming at improving specifically the Mode I behaviour and cohesive fracture, a combination with the PO material is investigated, which serves as an adhesion promoter between PI layers. The combination of the PO-layers sandwiched by PI-layers (material configuration 5) leads to significantly increased $G_{Ic}$ (by 144%) and $G_{IIc}$ (by 567%) values. Adhesive fracture between the PI and PO layer is observed for both Mode I and II (Figure 9).

In contrast, the set-up with an additional acrylate adhesive coating (material configuration 6) results in a reduction of 46% for Mode I, indicating that this additional bonding agent does not offer benefits regarding the interlaminar properties. In addition, the very thin layer configuration of only 25 μm in comparison to 125 μm of the other PI configurations might contribute to this negative effect. For material configuration 7, very low $G_{Ic}$ (only one valid test) and $G_{IIc}$ values have been identified. Crack propagation occurred in the PI layer under Mode I and in the PO layer under Mode II loading. The analysis of the experimental results indicate that the chosen crack initiation setup of embedding configuration II is considered disadvantageous, since a the crack propagation path is not pre-defined.

### 4.4. Embedding Configuration III

A combination of PO-layers sandwiched by PI-layers (material configuration 5) was identified as the most promising configuration in the previous experiments. However, it was also found that the crack initiation setup is subpar. Therefore, the effects of the PO adhesion promoter layer between PI layers on the delamiantion resistance are investigated in detail in the final phase of the investigations with an adapted crack initiation concept (Figure 1) and an alternative CFRP material. As discussed in Section 4.1, a qualitative comparability of the different material systems in terms of the delamination characteristics can be assumed.

Both set-ups of embedding configuration III, material configurations 8 and 9, show increased mode I energy release rates in comparison to the reference set-up, regardless of the position of crack initiation. In case of crack initiation between the PI and PO layer (material configuration 8), a very significant increase of 700% has been determined. Although the adhesive fracture pattern is similar to the one observed in material configuration 5, where the crack propagated between between the PI and PO layer also, the determined $G_{Ic}$ value of 2.27 kJ/m${}^{2}$ is about three times higher than the one of material configuration 5.

The trend of an improved delamination resistance can be also clearly observed for Mode II loading. For material configuration 8, the highest $G_{IIc}$ values with an increase of up to 989% are measured. However, it needs to be pointed out that the three highest values of the significantly increased results are based on experiments in which only one valid test was available for analysis.

## 5. Conclusions

The presented investigations deal with a detailed study on the influence of embedded functional layers to the mechanical behaviour of CFRP-composites. Different layers and composite lay-up configurations are investigated to identify reliable embedding configurations. The configurations are characterized using DCB and ELS tests to determine interlaminar delamination characteristics by comparing critical energy release rates for Mode I and Mode II loading conditions.

The Mode I results clearly indicate two groups of configurations: those leading to reduced interlaminar properties and crack opening resistance compared to the reference configuration and those with improved interlaminar behaviour. In contrast, all investigated configurations lead to increased $G_{IIc}$ values leading to the conclusion that embedded polymer layers foster a high Mode II crack propagation resistance.

Embedded PI layers exhibit the highest combined Mode I and II delamination resistance, where the observed cohesive fracture pattern seems to facilitate this effect. By utilizing PO adhesive promoter films, an enhanced Mode I delamination resistance is achieved. The adhesive capability of the PI layer with the CFRP and the ductility of the PO layer, which hinders crack propagation and bonds the PI layers, proved to be a very good combination with respect to Mode I interlaminar crack opening.

Both Mode I and Mode II results lead to the conclusion that the PI-PO-PI configuration is particularly effective for integrating a polymer film into high-performance CFRP-composite structures. It even leads to a reduction of delamination proneness. With this setup, the PI layers can be functionalized and additional electric elements such as sensors or actuators can be positioned between them. The PO layer serves as an internal electric insulation and also provides spacer and positioning capabilities. Electric insulation of the entire system to CFRP material is achieved by the PI-layers. As an outlook, this configuration is suggested to be transferred into further applications as ‘Tailored Embeddable Sensor and Actuator Layer (TEmSAL)’.

## Figures and Tables

**Table 1:** Investigated material and embedding configurations.

Material Configuration	Polymer Layer	Embedding Configuration	Layer Thickness [μm]	Crack Initiation between
1	PI	I	125	CFRP
2	PEEK	I	100	CFRP
3	PA	I	100	CFRP
4	PEI	I	100	CFRP
5	PI-PO-PI	II	350 (125 + 100 + 125)	CFRP
6	PI-PO-PI *	II	150 (25 + 100 + 25)	CFRP
7	PO-PI-PO	II	325 (100 + 125 + 100)	CFRP
8	PI-PO-PI	III	350 (125 + 100 + 125)	PI and PO
9	PI-PO-PI	III	350 (125 + 100 + 125)	PI and CFRP
10	none	reference	-	CFRP

* The PI layers are coated with an acrylate adhesive on the CFRP facing side.

**Table 2:** Specimen configurations.

Specimen	Dimensions [mm${}^{3}$]	Total Quantity (per Material Configuration)
DCB	277 × 25 × 3.8	40 (4)
ELS	277 × 25 × 3.8	40 (4)

**Table 3:** Determined Mode I and II energy release rates with the respective deviation from the reference configuration $\Delta_{ref}$.

Mat.	Polymer	Emb.	Mode I			Mode II		
Conf.	Layer	Conf.	$G_{\mathrm{Ic}}$ in kJ/m${}^{2}$	*S* in %	$\Delta_{\mathrm{ref}}$ in %	$G_{\mathrm{IIc}}$ in kJ/m${}^{2}$	*S* in %	$\Delta_{\mathrm{ref}}$ in %
1	PI	I	0.19	17	−32	2.78	22	281
2	PEEK	I	0 **	0	−100	0 **	0	−100
3	PA	I	0.05	69	−82	6.21 *	0	749
4	PEI	I	0.07	18	−76	5.68 *	0	677
5	PI−PO−PI	II	0.69	16	144	4.87	30	567
6	PI−PO−PI	II	0.15	5	−46	1.1	9	51
7	PO−PI−PO	II	0.05 *	0	−83	2.15	27	194
8	PI−PO−PI	III	2.27	4	704	7.96 *	0	989
9	PI−PO−PI	III	0.72	10	154	3.02	32	313
10	reference	ref.	0.28	13	0	0.73	10	0

* one valid test for analysis available; ** no measurable adhesion.

## Data Availability

The data presented in this study are available on request from the corresponding author.

## Abbreviations

The following abbreviations are used in this manuscript:

CFRP	Carbon Fibre Reinforced Plastic
DCB	Double-Cantilever Beam
DIC	Digital Image Correlation
ELS	End-Loaded Split
PA	Polyamide
PEEK	Polyether-Ether-Ketone
PEI	Polyetherimide
PI	Polyimide
PO	Polyolefine
PTFE	Polytetraflourethylene
UD	Unidirectionally Reinforced
